# RFRP Neurons Are Required for Acute Stress-induced Suppression of the Estrogen-stimulated LH Surge in Female Mice

**DOI:** 10.1210/endocr/bqaf106

**Published:** 2025-06-09

**Authors:** Maggie C Evans, Shaun M Stowe, India L Sawyer, Caroline Decourt, Frank Lee, Alexander S Kauffman, Greg M Anderson

**Affiliations:** Centre for Neuroendocrinology and Department of Anatomy, University of Otago School of Biomedical Sciences, Dunedin 9016, New Zealand; Centre for Neuroendocrinology and Department of Anatomy, University of Otago School of Biomedical Sciences, Dunedin 9016, New Zealand; Centre for Neuroendocrinology and Department of Anatomy, University of Otago School of Biomedical Sciences, Dunedin 9016, New Zealand; Centre for Neuroendocrinology and Department of Anatomy, University of Otago School of Biomedical Sciences, Dunedin 9016, New Zealand; Department of OBGYN and Reproductive Sciences and the Center for Chronobiology, University of California, San Diego, CA 92093, USA; Department of OBGYN and Reproductive Sciences and the Center for Chronobiology, University of California, San Diego, CA 92093, USA; Centre for Neuroendocrinology and Department of Anatomy, University of Otago School of Biomedical Sciences, Dunedin 9016, New Zealand

**Keywords:** RFRP, stress, corticosterone, LH surge, reproduction

## Abstract

The association between perceived stress and reproductive dysfunction is known, yet the underlying mechanisms remain incompletely determined. We previously demonstrated that RF-amide related (RFRP) peptide 3-expressing neurons, putative inhibitors of the central regulation of fertility, are required for both acute restraint stress- and glucocorticoid-induced suppression of LH pulsatility in female mice. The present study complemented this by testing the role of RFRP neurons in the stress-induced suppression of the estrogen-induced preovulatory-like LH surge. We first established a reliable model of acute restraint stress in mice that stimulates glucocorticoid secretion, suppresses a late afternoon estrogen-induced LH surge, and inhibits corresponding kisspeptin neuronal activation in the anteroventral periventricular brain region. Two hours of restraint stress initiated 2 to 6 hours prior to lights off met these criteria. We then ablated RFRP neurons in adult female mice by expressing a diphtheria toxin receptor specifically in these cells and exposing them to diphtheria toxin. RFRP neuron-ablated and control mice that were ovariectomized and estrogen-treated were exposed to the acute, mid-afternoon restraint stress protocol and assessed for their peak LH concentrations several hours later at the expected time of the LH surge. Control mice exhibited stress-induced suppression of the LH surge, as expected, whereas RFRP-ablated mice did not. No differences in peak LH concentrations were observed between nonstressed controls and stressed RFRP-ablated mice. These data suggest that acute psychosocial stress occurring several hours prior to preovulatory LH surge induction invokes RFRP neuron-mediated blockade of the surge. The neural circuitry involved remains to be fully characterized.

The hypothalamic GnRH neurons represent the final cumulative endpoint of a complex afferent neuronal system—termed the “GnRH neuronal network”—that integrates a multitude of regulatory signals to maintain optimal reproductive drive to the ovaries via the pituitary gonadotrophins, LH, and FSH. In females, estrogens are the primary feedback signal to this neuronal network, both for homeostatic negative feedback maintenance of tonic pulsatile GnRH/LH/FSH release as well as for the abrupt switch to positive feedback that drives a massive surge of GnRH/LH and consequent ovulation (1, 2). However, other hormonal, neuropeptide, and neurotransmitter modulators of GnRH also play important roles in regulating reproductive drive (3).

While reproductive function is required for species survival and thus physiologically safeguarded under normal conditions, in some cases, the integrated messages these modulators relay to the GnRH neurons is to adaptively suppress, or defer, reproductive function (4). For example, metabolically relevant hormones, such as leptin and insulin, are involved in integrating metabolic and reproductive function to ensure there is sufficient energy available to support reproduction (5). When there is insufficient energy availability to support the high energetic demands of pregnancy and lactation, it is advantageous to adaptively suppress and defer reproduction until conditions are more optimal. Similarly, activation of the hormonal stress axis—the hypothalamic-pituitary-adrenal (HPA) axis—releases glucocorticoid stress hormones that can also impact reproductive drive to ensure reproductive function is adaptively suppressed when conditions are suboptimal for rearing young.

While empirical data are limited, particularly in humans, both acute and chronic stress are known to be associated with reproductive suppression (6-8). Suppression of the preovulatory GnRH/LH surge represents a situation where even an acute stress exposure in the late follicular phase of the reproductive cycle could have relatively chronic fertility effects (9-11), due to the discrete timing of the LH surge and subsequent ovulation in females and the potential for missed conception opportunities. In mice, the LH surge occurs around 12 hours after the beginning of the light phase on the day of proestrus; this period represents a specific window during which stress could potentially prevent ovulation. The mechanisms underpinning stress-related reproductive dysfunction are not fully understood yet are often associated with activation of the HPA axis and glucocorticoid release. Indeed, corticosterone treatment to estrogen-primed female mice completely blocks their late afternoon LH surge (12), indicating that high levels of stress hormones are linked to impairment of the endocrine events triggering ovulation. However, glucocorticoids do not appear to directly target the GnRH neurons to a significant degree, as only a small fraction of GnRH neurons contain glucocorticoid receptors (13).

The RF-amide related peptide (RFRP) neurons located in the dorsomedial nucleus, which are part of the GnRH neuronal network, are both inhibitory to GnRH cell firing and downstream LH secretion (14-16) and contain abundant glucocorticoid receptor expression (17). RFRP neurons are thus suitably positioned to mediate stress-induced reproductive suppression. Furthermore, several lines of evidence suggest RFRP neurons mediate the suppressive effects of stress on the reproductive axis. First, both acute and chronic stress increase *Rfrp* gene expression in male and female rats (17, 18). This stress effect on RFRP neurons is blocked by adrenalectomy (17), highlighting glucocorticoids as critical mediators. Second, acute psychosocial stress increases *Rfrp* expression and RFRP neuron activation status (assessed by *cfos* induction) along with reduced LH pulse secretion in mice of both sexes (19, 20). Third, the inhibitory effect of fasting on LH secretion was partially reversed in RFRP-3 receptor (NPFFR1) knock-out mice (21). Fourth, knockdown of hypothalamic *Rfrp* prevented chronic stress-induced infertility and embryo resorption in rats (18). Last, our group previously showed that ablation or silencing of RFRP neurons prevented restraint stress or glucocorticoid-induced suppression of LH pulses (22) and that chemogenetic activation of RFRP neurons suppresses LH pulse frequency in female mice (23). While the suppression of pulsatile LH secretion can have profound effects on the subsequent preovulatory LH surge due to its role in supporting folliculogenesis and steroidogenesis, we were interested in determining whether the known inhibitory effects of acute stress on tonic LH pulsatility are similarly observed on the preovulatory LH surge. Therefore, in the present study, we determined whether RFRP neurons mediate acute stress-induced suppression of the preovulatory LH surge, which is required for ovulation and successful reproduction. The rodent GnRH/LH surge is known to be potently driven by the marked estrogen- and circadian-induced activation of a hypothalamic population of kisspeptin neurons located in the anteroventral periventricular nucleus (AVPV) (2, 24), and this activation of AVPV kisspeptin neurons can be completely suppressed by exposure to high levels of glucocorticoids (12). The present study therefore also investigated whether acute psychosocial stress impacts LH surge secretion indirectly via negative modulation of AVPV kisspeptin neuronal activity.

## Methods

### Animal Care

For experiment 1, adult female C57BL/6 mice purchased from Envigo were pair housed and studied at the University of California, San Diego (UCSD). For experiment 2, female offspring of *Rfrp*-IRES-Cre and floxed diphtheria toxin receptor breeder pairs (source and genetics described further later) were bred at the University of Otago animal breeding facility and were group housed for the experiment. All mice were maintained on a 12-hour light:12-hour dark cycle with lights on at 6 Am and maintained at a constant temperature (21 ± 1 °C), with ad libitum access to food and water. Experiment 1 was conducted at UCSD and performed in accordance with the National Institute of Health Animal Care and Use Guidelines and with authorization from the local Institutional Animal Care and Use Committee at UCSD. Experiment 2 was conducted at the University of Otago and was approved by the University of Otago Animal Ethics Committee.

### Experiment 1: Generating a Model of Acute Stress-induced Suppression of the Estradiol-induced LH Surge

#### Ovariectomies and estradiol implants

A well-established LH surge induction model was used in which exogenous estradiol (delivered subcutaneously via a custom-made continuous-release Silastic implant) drives daily preovulatory-like LH surges that peak just before the onset of the dark phase in ovariectomized female mice. Adult female C57BL/6 (9-12 weeks old) mice were anesthetized under isoflurane anaesthesia and bilaterally ovariectomized under sterile conditions. Mice were then immediately implanted subcutaneously with an estradiol implant that produces a high physiological level of circulating estradiol shown previously to evoke a large LH surge 48 hours later (24-27). The estradiol implants were made using 2.2-cm lengths of silicone tubing (1.98-mm inner diameter, 3.18-mm outer diameter, Silastic brand), and filled to 1.2-cm length with 17β estradiol solution (25 µL of 30 µg/mL, E8875, Sigma Aldrich) dissolved in sesame oil. Each capsule contained approximately 0.75 µg of 17β estradiol.

#### Treatment groups and acute stress induction

**Figure 1.**
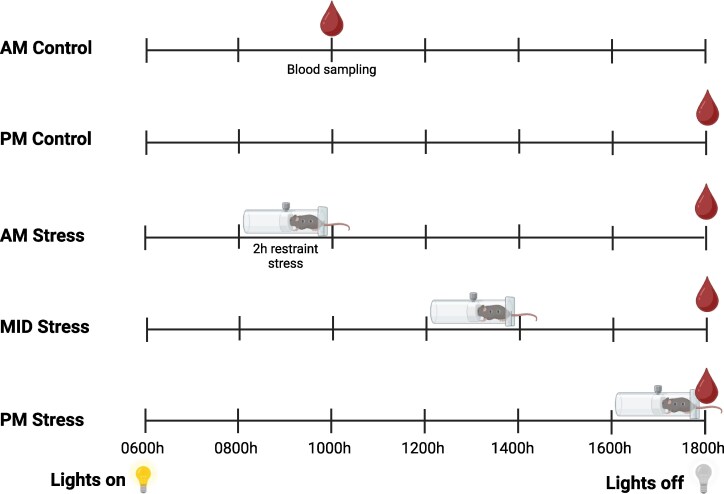
Schematic illustration of experiment 1. All mice underwent an estradiol-induced LH surge induction protocol. On the day of sampling they were divided into 5 experimental groups: (1) Am control mice did not receive any stress and their blood was sampled at 10 Am, (2) Pm control mice did not receive any stress and their blood was sampled at 6 Pm , (3) Am stress mice received 2 hours of restraint stress from 8-10 Am and their blood was sampled at 6 Pm, (4) MID stress mice received 2 hours of restraint stress from 12-2 Pm and their blood was sampled at 6 Pm, and (5) Pm stress mice received 2 hours of restraint stress from 4-6 Pm and their blood was sampled at 6 Pm. The estradiol-induced LH surge is circadian and occurs around the time of lights out, which is 6 Pm.

Ovariectomized, estrogen-primed mice were allocated to a stress treatment group at the time of their surgery, and the acute (2-hour) stress protocol and retro-orbital blood collection at sacrifice conducted 2 days later, on the day of the expected LH surge. The 5 treatment groups were as follows: (1) morning (Am) control (no stress, blood collected at 10:00 Am), (2) morning (Am) stress (stressed 8:00-10:00 Am, blood collection at 6:00 Pm), (3) midday (MID) stressed (stressed 12-2 Pm, blood collection at 6 Pm), (4) late afternoon (Pm) stress (stressed 4:00-6:00 Pm, blood collection at 6 Pm), and (5) late afternoon (Pm) control (no stress, blood collection at 6 Pm) (Fig. 1). “Stressed” mice received 2 hours of acute restraint stress during which time they were temporarily placed into Broome rodent restraint devices (Harvard Apparatus) in individual clean cages. At the conclusion of the 2 hours of restraint, stressed mice were returned to their home cage with their cage mate (that received the same stress protocol) until blood collection, or for the Pm stress group blood collection occurred immediately following restraint. No-stress control mice were not restrained and remained in their home cages with their cage mate until the time of blood collection. For blood collection, mice were briefly anesthetized with isoflurane and blood samples (∼300 µL) collected from the retroorbital plexus into a heparinized capillary tube, after which the mice were immediately culled. Blood was allowed to clot at room temperature for 90 minutes and then centrifuged for 15 minutes, after which serum was collected and frozen at −20 °C until used for LH, estradiol, and corticosterone concentration determination.

#### Hormone measurements

LH and estradiol were assayed by the Ligand Assay and Analysis Core (University of Virginia Center for Research in Reproduction). To determine LH concentration, 65-µL serum aliquots were assayed using a sensitive 2-site mouse LH sandwich radioimmunoassay (27). The assay limit of detectability was reported as 0.04 ng/mL. Using this specific LH assay, circulating LH concentrations of 0.7 ng/mL or above were considered surge values, as in our prior studies (28). This cutoff value was used to designate mice as surging or nonsurging to calculate the proportion of surging mice in each group. To determine estradiol concentration, 35 µL of serum was assayed using the Calbiotech ELISA (27), which has a reported assay sensitivity of 3 pg/mL. Only mice with estradiol levels above 13 pg/mL were determined to have reached circulating estradiol levels representative of pro-estrous mice and included in the results. On this basis, 3 mice were excluded from the study. All 5 groups had similar mean estradiol levels, with no significant statistical differences between groups (Amcontrol: 17.6 ± 1.7 pg/mL; Pm control: 18.1 ± 0.8 pg/mL; Am stress: 18.8 ± 2.4 pg/mL, MID stress 17.7 ± 1.9 pg/mL, Pm stress: 18.0 ± 1.3 pg/mL). To determine corticosterone concentration, 2.5 µL of serum was assayed in-house using the DetectX corticosterone enzyme immunoassay kit. The assay sensitivity averaged 2 ng/mL and the mean inter-assay and intra-assay coefficients of variation were <10%.

#### Double-label in situ hybridization for *cfos* expression in *Kiss1* neurons

Immediately following blood collection, brains were collected fresh frozen onto dry ice and stored at −80 °C. Frozen brains were sectioned on a cryostat into 5 alternating sets of 20-µm sections onto Superfrost-plus slides and stored at −80 °C. Double-label in situ hybridization was performed using our laboratory's standard protocol as previously described (24, 27, 29, 30) to determine the degree of *Kiss1* and *cfos mRNA* coexpression (a measure of neuronal activation) in the hypothalamic AVPV region. Briefly, 1 set of slide-mounted brain sections encompassing the AVPV region was fixed in 4% paraformaldehyde, pretreated with acetic anhydride, rinsed in 2X SSC, delipidated in chloroform, dehydrated in ethanols, and air-dried. Radio-labeled (^33^P) antisense *cfos* (0.05 pmol/mL) and digoxigenin (DIG)-labeled *Kiss1* riboprobes (Roche Digoxigenin labeling kit, 1:500) were combined with tRNA, heat denatured, dissolved together in hybridization buffer, applied to slides (100 μL/slide), and hybridized at 55 °C overnight. The next day, slides were washed in 4X SSC and then treated with RNAse A at 37 °C, and subsequently washed in 0.1X SSC at 62 °C. Slides were then incubated in 2X SSC with 0.05% Triton X-100 containing 3% normal sheep serum for 75 minutes at room temperature and then incubated overnight at room temperature with anti-DIG antibody conjugated to alkaline phosphatase (Roche; 1:500). The next day, slides were washed and then incubated with Vector Red alkaline phosphatase substrate (Vector Labs, CA) for 1 hour at room temperature. Slides were then air-dried, dipped in Kodak NTB emulsion, stored at 4 °C, and then developed and cover-slipped. ISH slides were analyzed “blindly” with respect to treatment using microscopy linked to an automated imaging processing system (Dr. Don Clifton, University of Washington) that identifies and counts the number of red DIG-containing (*Kiss1* mRNA) cells under fluorescence microscopy and then quantifies the number of silver grains (*cfos* mRNA) overlying each DIG cell under dark-field microscopy. A minimum of 3 AVPV sections and 75 *Kiss1* cells was counted for each animal. Signal-to-background ratios for individual cells were calculated by the program and a cell considered double-labeled if its ratio was >3, as in prior studies (24, 28).

### Experiment 2: Stress-induced Suppression of the Estradiol-induced LH Surge Requires Intact RFRP Neurons

#### Generation of RFRP-ablated female mice

To assess the functional role of RFRP neurons for stress-induced LH surge suppression, RFRP neurons were permanently ablated in adult female mice. Heterozygous *Rfrp*-IRES-Cre mice (B6(Cg)-*Npvf^tm1.1(icre)Gand^*, https://www.informatics.jax.org/strain/MGI:7439911) (22) were crossed with mice homozygous for Cre-dependent diphtheria toxin receptor (DTR) expression (Gt(ROSA)26Sor^tm1(HBEGF)Awai^/J, The Jackson Laboratory, RRID:IMSR_JAX:007900) (31), as described previously (22). In this model, the inducible DTR is only expressed in cells that ever-expressed Cre recombinase, which in this case is only RFRP neurons. Thus, in our *Rfrp*-IRES-Cre+/DTR floxed mice, only RFRP cells are susceptible to ablation by acute diphtheria toxin treatment, whereas all other cells are unaffected. All female mice were genotyped from tail tip DNA. The *Rfrp*-IRES-Cre line was genotyped using generic Cre primers (forward: 5′-CCT GGA AAA TGC TTC TGT CCG-3′; reverse: 5′-CAG GGT GTT ATA AGC AAT CCC-3′; annealing temperature 55 °C; product size indicating the Cre allele: 392 bp). Floxed diphtheria toxin receptor mice were identified using the following primers, 5′-AAA GTC GCT CTG AGT TGT TAT-3′ (common forward primer), 5′-GGA GCG GGA GAA ATG GAT ATG-3′ (wild-type reverse primer), and 5′-CAT CAA GGA AAC CCT GGA CTA CTG-3′ (mutant reverse primer); annealing temperature 61 °C; product size indicating the floxed and wild-type alleles: 603 and 242 bp, respectively. To induce RFRP neuronal ablation, adult (10-12 weeks old) mice received a single injection of diphtheria toxin (0.05 mg/kg subcutaneously in 200 µL of saline) and allowed 4 weeks for sufficient neuronal ablation to take place, before being used for the experiment 2 restraint stress study. Control mice included Cre/DTR floxed females similarly treated with DT.

#### Treatment groups and acute stress induction

For experiment 2, a well-established variation of the LH surge induction model described in experiment 1 was used in which low-dose exogenous estradiol (delivered via a custom-made continuous-release subcutaneous implant) maintains negative feedback for 6 days and a preovulatory-like LH surge is then generated around the onset of the dark phase in response to an injection of estradiol benzoate (32-34). Different LH surge induction models and LH assays were used in experiment 1 vs experiment 2 because the 2 experiments were conducted at different research sites, and each site used the respective protocol with which they had the most experience. To this end, all RFRP-ablated and control female mice in experiment 2 were ovariectomized and implanted subcutaneously with estradiol implants (1.0-mm internal diameter, 2.1-mm external diameter, Silastic brand silicone rubber containing ∼1 µg 17β estradiol in Silastic brand Medical grade A silicone adhesive) that produce low physiological levels of estradiol designed to maintain steroid negative feedback. Six days later, mice were given a subcutaneous injection of β-estradiol 3-benzoate (1 µg in 100 µL in sesame oil; Sigma-Aldrich) at 9 Am, as described previously (32), to induce an LH surge. On the following day, a basal tail tip blood sample (4 µL diluted immediately in 150-µL PBS) was then taken from all mice and immediately placed on dry ice and then stored at −20 °C until required. At 2 Pm, RFRP-ablated and control mice (n = 5 per group) were removed from their home cages and exposed to 2 hours of restraint stress by being individually placed in a plastic restraint cone (Decapitone; Braintree Scientific) in a new cage. This period was selected as the mid-point of the 2 restraint treatments that were effective in suppressing the LH surge and inhibiting AVPV Kiss1 neuron activation in experiment 1. The “no stress” control mice from each group remained in their home cages. Tail tip blood samples were again taken from all mice at 4 Pm, 5 Pm, and 6 Pm, the latter time being the expected time of the LH surge.

#### Validation of RFRP neuronal ablation

To confirm successful RFRP-3 neuronal ablation in diphtheria toxin-treated RFRP-Cre+ mice, at the end of the experiment (∼6 weeks after diphtheria toxin injection), a subset of the experimental mice (6 RFRP-ablated and 7 controls) was anesthetized with 250 mg/kg sodium pentobarbital and perfused through the heart with 4% PFA in 0.1 mL PBS, pH 7.4. Brains were removed, postfixed in PFA, and cryoprotected in 30% sucrose solution. Coronal (30-µm thick) sections from the dorsomedial hypothalamus (DMH), where all RFRP neurons reside, were cut from each brain on a sliding microtome with a freezing stage to provide 3 sets of consecutive sections (90 µm apart). One series of brain tissue from each animal was then used to label for RFRP-3-immunoreactive cells using nickel-enhanced 3,3′-diaminobenzidine labelling (22). All immunohistochemistry steps were performed at room temperature unless noted otherwise and were separated by 4 washes in 0.05 M Tris-buffered saline. Sections were blocked in 0.25% BSA made up in Tris-buffered saline containing 0.5% Triton-X for 20 minutes and incubated for 24-48 hours at 4 °C in polyclonal rabbit anti-sparrow GnIH (an RFRP-3 ortholog) (PAC 123a, kindly provided by Dr George Bentley, University of California Berkeley; 1:5000 dilution; RRID: AB_ 2531898). This was followed by incubation in 1:1000 biotinylated goat anti-rabbit (Vector Laboratories, RRID:AB_2313606) for 1 hour, in Vector Elite avidin-biotin complex solution (Vector Laboratories) for 1 hour and in nickel-enhanced 0.5% 3,3′-diaminobenzidine and hydrogen peroxide solution (Sigma Millipore) for 5 to 7 minutes until a blue-black staining was observed. The stained sections were mounted on slides, dehydrated, and coverslipped using DPX mounting medium. All RFRP soma from at least 4 sections per animal were examined at 200× magnification using an Olympus BX45 microscope.

#### Hormone measurement

Whole blood LH concentrations in experiment 2 were assessed in duplicate 50-µL aliquots using a sensitive in-house sandwich ELISA, as previously described (35). The assay sensitivity averaged 0.1 ng/mL, and the interassay and intra-assay coefficients of variation were <10%. An LH concentration of >2 ng/mL was considered a surge in this assay, as in prior studies (33). Serum corticosterone concentration was assayed as described for experiment 1. The assay sensitivity averaged 4 ng/mL and the interassay coefficient of variation was <10%.

#### Statistical analysis

Statistical analyses were performed and data graphed using Prism software 9.0 (GraphPad). All data are graphed and presented in text as mean ± SEM. The proportion of each group exhibiting an LH surge in experiment 1 was analyzed using a Fisher exact test. For all other data, a Student *t*-test was used to compare 2 groups; a 1-way ANVOA with a Holm-Šídák multiple comparisons post hoc analysis was used to compare the effects of acute restraint stress at different times of day (experiment 1), and a 2-way ANOVA with a Holm-Šídák post hoc analysis was used to compare the effects of 2 factors (acute restraint stress and RFRP neuronal ablation; experiment 2). A *P* value <.05 was considered statistically significant. Group sizes are reported in the figure legends.

## Results

### Generating a Model of Acute Stress-induced Suppression of the Estradiol-induced LH Surge

**Figure 2.**
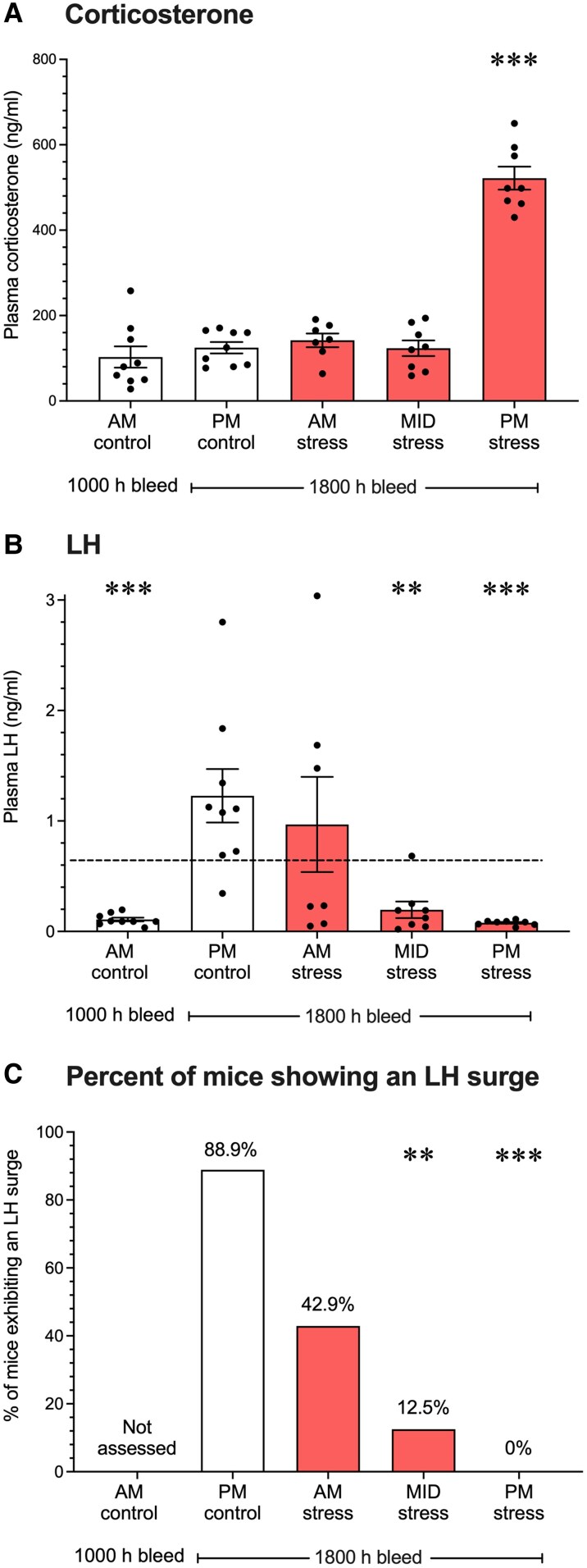
Acute afternoon restraint stress blocks the LH surge and transiently elevates corticosterone secretion. (A) Corticosterone response in female control (white bars) or acute restraint stress-exposed (red shaded bars) mice. (B) Average LH concentration in the same plasma samples as those for panel A. (C) Percentage of mice exhibiting an LH surging above the 0.7 ng/mL threshold. Data are mean ± SEM, analyzed using a 1-way ANOVA with Holm-Šídák multiple comparison testing relative to PM controls. n = 7-9 mice per group. The horizontal dashed line in panel B indicates the threshold value for an LH surge in experiment 1, using the LH sandwich radioimmunoassay. **Significantly different from Pm controls; *P* < .01; ***significantly different from Pm controls; *P* < .001.

Before testing the hypothesis that RFRP neurons are critical for suppression of the LH surge in response to acute stress, we first determined the best time window to administer acute (2 hours) restraint stress to stimulate glucocorticoid release and suppress the late afternoon estradiol-induced LH surge. To this end, mice were exposed to 2 hours restraint stress in either the (1) morning (Am stress, 8-10 Am), (2) midday (MID stress, 12-2 Pm), or (3) late afternoon (Pm stress, 4-6 Pm), and blood samples taken at the predicted time of the estradiol-induced LH surge (6 Pm; assayed for both LH and corticosterone) (1). Blood samples were also taken from “no stress” control groups in the morning (Am control, 10 Am; no surge expected) and late afternoon (Pm control, 6 Pm; surge expected) (Fig. 1). There was a significant overall effect on corticosterone concentration between groups (F(_4, 36_) = 73.3; *P* < .001; Fig. 2A). Holm-Šídák multiple comparison tests revealed that the corticosterone levels of Am and Pm nonstress control groups and the Am and MID stress groups were not significantly different vs one another, suggesting that the Am and MID stress groups had each recovered from their prior 2-hour bout of restraint stress in terms of HPA axis activity at the time of blood sampling at 6 Pm. Only the PM stress group, which was undergoing acute restraint stress up until the blood sample was taken at 6 Pm, still exhibited high levels of circulating corticosterone (*P* < .001 vs Pm control mice; Fig. 2A). This demonstrates that our 2-hour restraint stress paradigm greatly activates the HPA stress axis.

We next determined whether 2 hours of restraint at each of the different time periods (Am, MID, Pm) was sufficient to suppress a late afternoon estradiol-induced LH surge. An overall effect was found between groups (F(_4,36_) = 7.2; *P* < .001; Fig. 2B). As expected, the PM no-stress control group had significantly higher LH levels, indicative of an LH surge, compared to the AM no-stress control group, the latter of whose blood was collected in the morning (10 Am) when the surge does not occur (*P* < .001; Fig. 2B). The MID stress and Pm stress groups both had significantly lower mean LH levels than the Pm nonstress controls, indicative of suppressed LH surges in those 2 stress groups (Pm control vs MID stress, *P* = .002; Pm control vs Pm stress, *P* < .001; Fig. 2B). There was no difference in the mean LH concentration between Pm no-stress control mice and Am stress mice (*P* = .39).

We also calculated the percentage of mice showing an LH surge in each of the 4 groups for which blood was collected in the Pm period (when the surge normally occurs). Only 1 of the 8 MID-stress mice and none of the 8 Pm-stress mice demonstrated an LH surge, whereas nearly all (8 of 9) of the Pm nonstress controls showed a surge. Indeed, the Pm control group had a significantly greater proportion of surging mice (88.9%) compared to the MID-stress (12.5%) and Pm-stress (0%) groups but not compared to the Am stress group, which showed a mixed LH surge response (42.9% surging) (Pm control vs MID stress, *P* = .0034; vs Pm stress, *P* = .0004; vs Am stress, *P* = .153; Fig. 2C). Based on these outcomes, we concluded that a 2-hour bout of restraint stress initiated any time between 12 Pm and 4 Pm is sufficient to suppress the estradiol-induced LH surge later that afternoon (lights off at 6 Pm). For the next study, which tested the role of RFRP neurons (experiment 2), a restraint stress exposure time of 2 Pm to 4 Pm was therefore selected.

**Figure 3.**
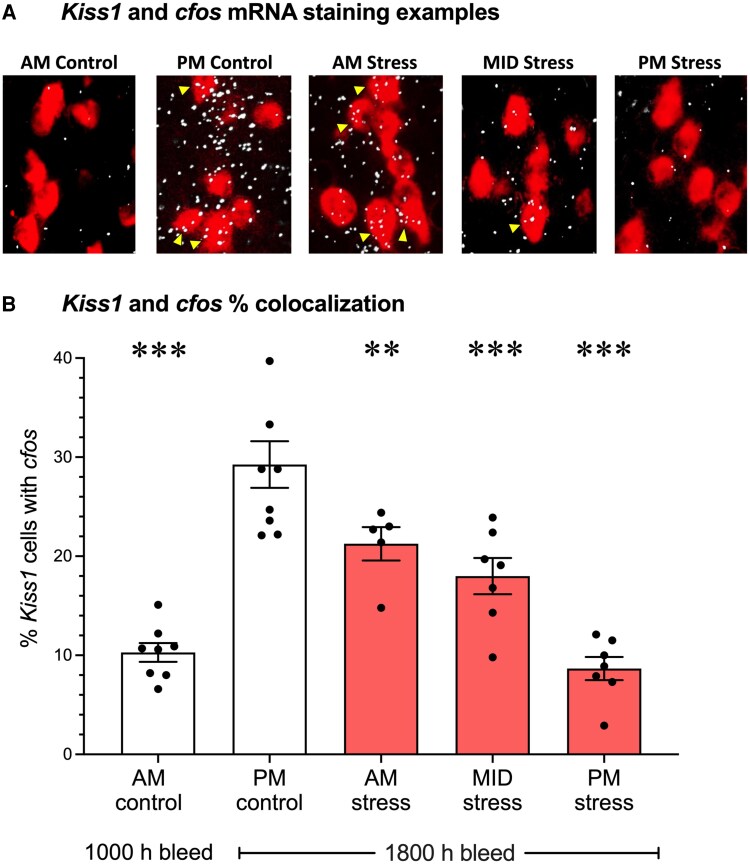
Acute stress inhibits AVPV *Kiss1* neuronal activation status. (A) Representative images of double label in situ hybridization staining for *Kiss1* mRNA (red cytoplasmic fluorescence) and *cfos* mRNA (white silver grains) in the hypothalamic AVPV of female mice. Yellow triangles denote examples of co-labeled neurons. (B) Mean percentage of AVPV *Kiss1* neurons coexpressing *cfos* mRNA in female control (white bars) or acute restraint stress exposed (red shaded bars) mice. Data are mean ± SEM, analyzed using a 2-way ANOVA with Holm-Šídák multiple comparison testing relative to Pm controls. n = 5-8 mice per group. **Significantly different from Pm controls; *P* < .01; ***significantly different from Pm controls; *P* < .001.

We next determined whether the observed inhibition of LH surges in the stressed mice reflected an inhibition of the neuronal activation of AVPV kisspeptin neurons, which are implicated as key drivers of the oestradiol positive feedback induction of LH surges (2, 36) and known to be inhibited by stress hormones (12). We therefore examined *cfos* mRNA induction in AVPV *Kiss1* neurons (a measure of *Kiss1* neuronal activation) in control and stressed females at the time of blood and brain collection (Fig. 3A). Am control mice, which had low nonsurging levels of LH, exhibited low *cfos/Kiss1* coexpression, whereas Pm control females, with high surge levels of LH, demonstrated a significantly higher degree of *cfos/Kiss1* coexpression (*P* < .001; Fig. 3B), indicating a large proportion of their AVPV *Kiss1* neurons were activated, as expected. By contrast, 2 hours of restraint stress at either Am, MID, or Pm time-points was sufficient to significantly inhibit *Kiss1* neuronal activation levels compared to Pm controls (*P* < .01 vs Am and MID stress, *P* < .001 vs Pm stress; Fig. 3B). *Kiss1 + cfos* coexpression was not significantly different between the Pm stress and Am control groups, and both were significantly lower than the Am stress and MID stress groups (*P* < .001 vs Am stress; *P* < .01 vs MID stress, Fig. 3B). There was no difference in mean *Kiss1* neuron activation between Am and MID stress groups (*P* = .239).

### Experiment 2: Stress-induced Suppression of the Estradiol-induced LH Surge Requires Intact RFRP Neurons

#### Validation of RFRP-ablated mice

**Figure 4.**
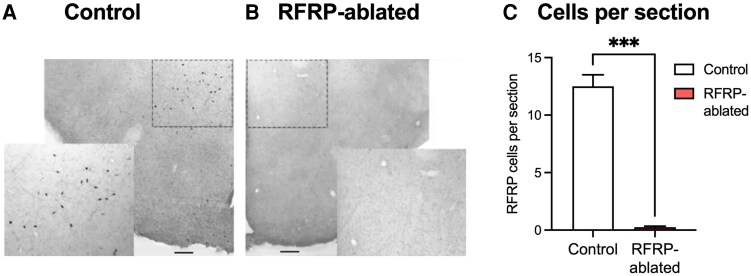
Validation of RFRP-ablated mice. Representative images of coronal sections from the dorsal medial nucleus of the hypothalamus showing RFRP-3 immunolabelling in adult control (A) and RFRP-ablated (B) female mice. The number of cells per section is shown in panel C. Data are mean ± SEM, analyzed using a Student *t*-test. n = 10 mice per group. Scale bars indicate 200 μm. ***P < .001.

After having validated a model of acute stress-induced suppression of the LH surge in experiment 1, we next tested our hypothesis that RFRP neurons play a critical role mediating the effects of stress to the HPG axis during the preovulatory LH surge. To this end, we crossed RFRP-Cre positive mice with Cre-dependent DTR-expressing mice to ultimately generate, after acute adulthood diphtheria toxin treatment, RFRP-ablated mice and littermate controls. As shown in the representative image in Fig. 4A, control mice exhibited abundant RFRP-3 immunoreactive cells in the dorsomedial nucleus (12.3 ± 1.12 neurons per section), as expected, whereas essentially no RFRP-3 immunoreactivity was observed in RFRP-ablated mice (0.1 ± 0.06 neurons per section; *P* < .001; Fig. 4B). We have previously characterized RFRP-ablated mice, showing they exhibit no obvious differences in estrous cyclicity or reproductive organ weight under normal, nonstressed conditions (22).

#### Stress-induced suppression of the estradiol-induced LH surge requires intact RFRP neurons

**Figure 5.**
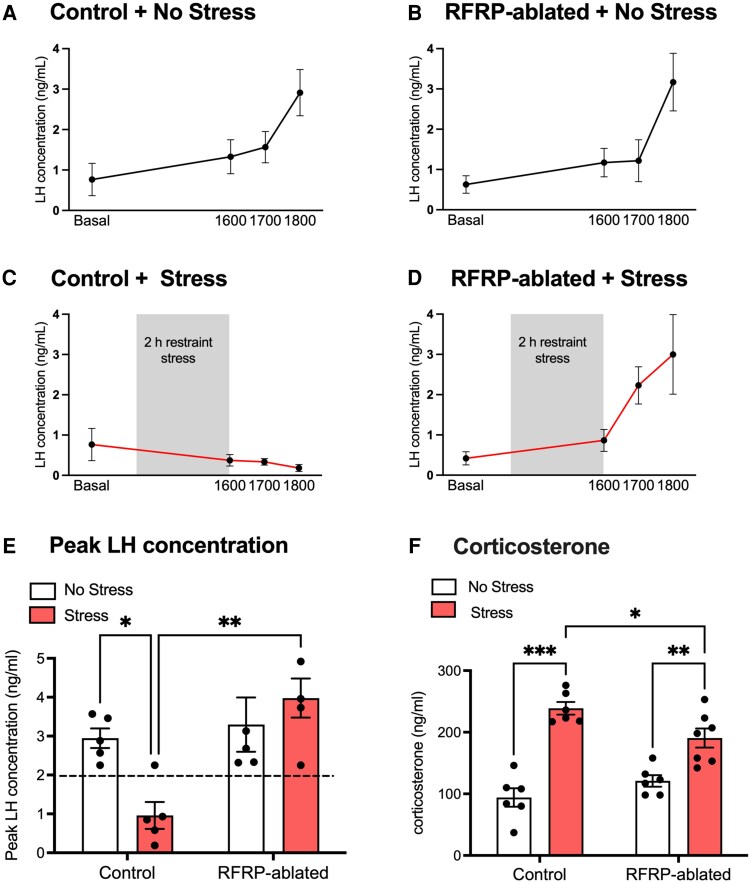
RFRP neuronal ablation precludes stress-induced LH surge suppression. Surge-like LH profiles in nonstressed control (A) and RFRP-ablated (B) mice at 6 Pm. (C) Absence of surges in control mice exposed to 2 hours of acute restraint stress from 2-4 Pm. (D) Full magnitude surges in RFRP-ablated mice exposed to 2 hours of acute restraint stress from 2-4 Pm. (E) Peak LH concentration was significantly reduced in control mice exposed to acute restraint stress compared to all other groups, whereas peak LH was not suppressed by restraint stress in RFRP-ablated mice. (F) Blood corticosterone concentrations at 4 Pm were significantly elevated by restraint stress in both groups, albeit to a slightly lesser extent in RFRP-ablated mice than in controls. The horizontal dashed line in panel E indicates the threshold value for an LH surge in experiment 2 using the LH ELISA assay. Data are mean ± SEM, analyzed using a 2-way ANOVA with Holm-Šídák multiple comparison testing. n = 5-7 mice per group. *P < .05; **P < .01; ***P < .001.

To test whether RFRP neurons mediate the suppressive effects of acute stress on the LH surge, we exposed ovariectomized, estrogen-treated RFRP-ablated, and littermate control mice to 2 hours of restraint stress from 2 Pm to 4 Pm and then assessed whether they exhibited an LH surge before lights off. As additional controls, we also included an RFRP-ablated group and an RFRP-intact control group that were not exposed to any stress. We analyzed LH levels in stressed and no-stress RFRP-ablated mice and controls. As shown in Fig. 5A and 5B, in the absence of any stress exposure, both the control and RFRP-ablated mice exhibited pronounced estradiol-induced LH surges at 6 Pm (serum concentration >2 ng/mL in this assay) (33), as expected. In contrast, only 1 of the control mice exposed to 2 hours of restraint stress exhibited an estradiol-induced LH surge (Fig. 5C), consistent with what we previously observed in experiment 1. In contrast, 100% of the RFRP-ablated mice exposed to 2 hours of restraint stress exhibited surge levels of LH at 6 Pm (Fig. 5D). A main effect of stress (F(_1, 16_) = 12.29; *P* = .003), but not group (F(_1, 16_) = 1.84; *P* = .193) was observed in peak LH concentration. A significant interaction was observed (F(_1, 16_) = 7.71; *P* = .013), indicating the RFRP-ablated mice responded differently to acute restraint stress than control mice (Fig. 5E). Accordingly, no differences in peak LH concentration were observed between the 2 “no stress” groups and the stressed RFRP-ablated group, whereas the stressed control mice showed significantly blunted peak LH concentrations vs the “no stress” controls (*P* = .039) and the stressed RFRP-ablated group (*P* = .0024).

A main effect of stress (F(_1, 21_) = 66.6; *P* < .001), but not group (F(_1, 21_) = 0.66; *P* = .423) was observed in serum corticosterone concentrations at 1600 hours. Corticosterone levels were significantly elevated by stress exposure in both of the restraint stress groups (controls: *P* < .001; RFRP-ablated: *P* = .003), indicating that ablating RFRP neurons does not prevent a rise in corticosterone during acute stress. A significant interaction was observed (F(_1, 21_) = 8.18; *P* = .009), indicating the RFRP-ablated mice responded differently to acute restraint stress than control mice. While there was no difference in corticosterone level between the 2 “no stress” groups, corticosterone levels were slightly more elevated in the stressed controls compared to the stressed RFRP-ablated mice (*P* = .030) (Fig. 5F), though this was not a large magnitude difference between the 2 groups. These data support our hypothesis that RFRP neurons are required for acute restraint stress-induced suppression of the preovulatory LH surge.

## Discussion

The neuroendocrine control of reproductive function in mammals is complex, integrating a multitude of inputs to adapt to ever changing external environmental and internal physiological conditions. One of the least understood interactions is that between emotional stress and infertility, which has been documented for decades but has proven difficult to tease out empirically (37). After first validating a model of acute stress-induced reproductive suppression, we functionally investigated the role of the putative inhibitory RFRP neurons in the regulation of the estradiol-induced LH surge in response to acute stress. In keeping with the putative inhibitory role for RFRP-3 on the HPG axis, our results demonstrate that RFRP neurons are critically required for mediating the negative effects of perceived acute stress on the preovulatory LH surge in mice. In control female mice with their RFRP neurons intact, 2 hours of restraint stress was sufficient to completely block the late afternoon estradiol-induced LH surge. In stark contrast, in female mice whose RFRP neurons were ablated, 2 hours of restraint stress was unable to block the estradiol-induced LH surge, even though such stress exposure still significantly increased circulating corticosterone in these females. While RFRP neurons do not appear to play a critical role in the regulation of mammalian reproduction under normal nonstressed conditions, our findings build on the accumulating evidence (17-20, 22) that these neurons play a central role in the mechanism whereby perceived psychosocial stress is relayed to—and inhibits—the neuroendocrine reproductive axis.

Chronic corticosterone treatment has been previously shown to potently inhibit the LH surge in estradiol-treated female mice, possibly by inhibiting the AVPV kisspeptin neurons (12), which also express the RFRP-3 receptor *Npffr1* and are direct targets of RFRP neurons (38). Such inhibition of AVPV kisspeptin neurons during corticosterone exposure suggests that acute or chronic stress paradigms that elevate corticosterone may also block the LH surge via upstream direct or indirect inhibition of AVPV neurons. We have shown that the AVPV kisspeptin neuronal population begins to increase its activity (both *Kiss1* mRNA levels and *cfos* coexpression) at least 2 to 3 hours prior to the peak of the LH surge (24). The 2-hour periods of stress exposure in experiment 1 that occurred between 12 Pm and 6 Pm (MID and Pm stress), which suppressed LH surge activity in the vast majority of mice, would be expected to overlap with this timing of increasing kisspeptin neuronal activity. In contrast, the acute stress occurring between 8 and 10 Am (Am stress) may have preceded the time of the LH surge onset by a long enough duration that nearly half the stressed mice were able to successfully activate the GnRH neuronal network surge mechanism unimpeded. The ability of an acute stressor to exert a delayed effect of several hours on LH surge occurrence in some individuals demonstrates how even a brief stressful event in the late follicular phase could be capable of preventing ovulation from occurring. We postulate that RFRP neurons play a key role in indirectly “gating” the effects of glucocorticoids and/or cognitive stress signals on AVPV kisspeptin neurons, and in turn the GnRH neuronal activity that drives the LH surge. Future detailed studies characterizing this interaction and the mechanistic pathways involved are warranted. The present LH surge data build on our previous findings demonstrating that RFRP neuronal ablation prevents restraint stress-induced suppression of LH pulsatility in female mice (22), and that chemogenetic activation of RFRP neurons suppresses LH pulse frequency in female mice (23). Taken together, our former and present data suggest that RFRP neurons play a critical role mediating inhibitory effects of perceived acute stress to GnRH neurons during both phases of the female reproductive cycle—the maintenance of homeostatic negative feedback of tonic pulsatile GnRH/LH release and the switch to estrogen positive feedback that drives a surge of GnRH/LH and consequent ovulation.

RFRP neuronal activation has been shown to stimulate glucocorticoid release (22), which, as mentioned previously, is known to act centrally to suppress LH pulse frequency and delay/block the preovulatory LH surge (39). Therefore, in the absence of RFRP neurons, it is possible that a lack of RFRP-induced glucocorticoid release could explain the preserved estradiol-induced LH surge observed in RFRP-ablated mice poststress. The results from experiment 2 provide some insight into this. Corticosterone levels were significantly elevated in both stressed RFRP-ablated and stressed control mice, vs nonstressed counterparts, indicating that ablation of RFRP neurons does not prevent an acute stress-induced rise in circulating serum corticosterone. While the elevation in corticosterone concentration in response to 2 hours of restraint stress was slightly lower in RFRP-ablated mice than in controls, this difference was not a large magnitude and indeed, we have previously shown that a more acute (30-minute) period of restraint in RFRP-ablated female mice induced a rise in corticosterone that was not significantly different than that observed in RFRP-intact controls (22). It seems unlikely that a subtle reduction in stress-induced corticosterone levels would be sufficient to allow a robust LH surge to occur in 100% of RFRP-ablated mice. Therefore, we postulate that the preserved estradiol-induced LH surge observed in the RFRP-ablated mice poststress in the present study is more likely to be due to the absence of RFRP neurons and their downstream inhibitory effects on kisspeptin and GnRH neuronal activity. Whether these inhibitory effects of RFRP neurons on kisspeptin or GnRH neurons are occurring directly or indirectly remains to be determined, as does the specific signalling factors mediating this inhibitory action (eg, RFRP-3 vs other co-released signalling peptides or neurotransmitters).

We previously reported that RFRP-ablated female mice exhibit normal estrous cyclicity under nonstressed conditions (22). For testing whether RFRP neurons are required for stress-induced LH surge suppression, we decided against using ovary-intact, cycling females since robust proestrus surges are easily disrupted even by investigator handling stress in cycling mice, making it potentially difficult to distinguish the effects of restraint stress from daily handling stress (40). We chose instead to assess the effects of acute stress using well-described experimental estradiol-induced LH surge models. The switch from negative to positive estrogen feedback that drives the natural preovulatory LH surge is primarily driven by estrogen actions on the anteroventral periventricular kisspeptin neurons, and this mechanism appears to operate identically in both intact cycling mice and ovariectomized, estradiol-primed mice (40). Moreover, interpretation of stress effects on the preovulatory surge in cycling mice can be complicated by the difficulty in determining whether effects are due to direct inhibition of central estrogenic positive feedback mechanisms vs a disruption of tonic LH pulses that are needed to drive sufficient ovarian estradiol production for inducing the LH surge (9, 39). Indeed, we and others have shown that acute restraint stress can negatively affect LH pulsatility (9, 19, 22, 41), which in turn would diminish endogenous ovarian estradiol production, and absence of RFRP neurons negates this effect (22). Therefore, to directly test LH surge capability, our present experimental paradigms avoided this issue and permitted us to study whether the brain's surge system can still generate an LH surge in response to sufficient estradiol exposure in females that are acutely stressed.

While the present results support our hypothesis that RFRP neurons play a critical central role mediating perceived acute stress-induced anovulation, it remains unclear whether these neurons likewise mediate inhibitory effects of chronic psychosocial stress or the effects of acute or chronic immune and metabolic stress. In addition to glucocorticoids, there are multiple other stress-related hormones and signaling factors associated with these differing types of stressors, and therefore, presumably multiple central mechanisms may underly stress-induced anovulation and infertility (8). However, it remains likely that RFRP neurons play a key role in mediating the effects of any stress condition to the central reproductive axis when glucocorticoids are critically involved, since prior evidence indicates that RFRP ablation prevents the inhibitory effects of glucocorticoids on GnRH/LH pulse secretion (22). If so, there are several important avenues of future investigation to further identify pathways and mechanisms involved, including assessing the ability of exogenous corticosterone to block the LH surge in RFRP-ablated mice and testing whether RFRP neurons play a role upstream or parallel of AVPV kisspeptin neurons during acute stress exposure inhibition of LH surges.

In summary, our data support the hypothesis that acute psychosocial stress several hours prior to ovulation can sufficiently activate the HPA axis and subsequent corticosterone release, which in turn may act centrally via or in concert with RFRP neurons, presumably via RFRP-3 release, to block the preovulatory LH surge in mice. Future studies are needed to test the specific mechanisms and neural circuitry involved in this proposed model. The present results build on previous data (17, 18, 22) and further establish RFRP neurons as key allostatic modulators of reproductive function (42).

## Data Availability

Some or all datasets generated during and/or analyzed during the current study are not publicly available but are available from the corresponding author on reasonable request.

## Abbreviations

AVPV: anteroventral periventricular nucleus
DIG: digoxigenin
DTR: diphtheria toxin receptor
HPA: hypothalamic-pituitary-adrenal
MID: midday
RFRP: RF-amide related
UCSD: University of California, San Diego

## References

[1] Czieselsky K, Prescott M, Porteous R, et al Pulse and surge profiles of luteinizing hormone secretion in the mouse. Endocrinology. 2016;157(12):4794‐4802. 27715255 10.1210/en.2016-1351

[2] Kauffman AS . Neuroendocrine mechanisms underlying estrogen positive feedback and the LH surge. Front Neurosci. 2022;16:953252. 35968365 10.3389/fnins.2022.953252 PMC9364933

[3] Plant TM . 60 YEARS OF NEUROENDOCRINOLOGY: the hypothalamo-pituitary-gonadal axis. J Endocrinol. 2015;226(2):T41‐T54. 25901041 10.1530/JOE-15-0113 PMC4498991

[4] Carrasco RA, Breen KM. Allostasis in neuroendocrine systems controlling reproduction. Endocrinology. 2023;164(10):bqad125. 37586095 10.1210/endocr/bqad125 PMC10461221

[5] Evans MC, Anderson GM. Neuroendocrine integration of nutritional signals on reproduction. J Mol Endocrinol. 2017;58(2):R107‐R128. 28057770 10.1530/JME-16-0212

[6] Ferin M . Clinical review 105: stress and the reproductive cycle. J Clin Endocrinol Metab. 1999;84(6):1768‐1774. 10372662 10.1210/jcem.84.6.5367

[7] Geraghty AC, Kaufer D. Glucocorticoid regulation of reproduction. Adv Exp Med Biol. 2015;872:253‐278. 26215998 10.1007/978-1-4939-2895-8_11

[8] McCosh RB, Breen KM, Kauffman AS. Neural and endocrine mechanisms underlying stress-induced suppression of pulsatile LH secretion. Mol Cell Endocrinol. 2019;498:110579. 31521706 10.1016/j.mce.2019.110579 PMC6874223

[9] Dobson H, Tebble JE, Phogat JB, Smith RF. Effect of transport on pulsatile and surge secretion of LH in ewes in the breeding season. J Reprod Fertil. 1999;116(1):1‐8. 10505050 10.1530/jrf.0.1160001

[10] Norman RL, McGlone J, Smith CJ. Restraint inhibits luteinizing hormone secretion in the follicular phase of the menstrual cycle in rhesus macaques. Biol Reprod. 1994;50(1):16‐26. 8312440 10.1095/biolreprod50.1.16

[11] Roozendaal MM, De Kruijf HF, Reuling RJ, et al Inhibition of the LH surge by restraint stress in cyclic rats: studies on the role of GABAA and GABAB receptors. Stress. 1997;1(4):241‐248. 9787248 10.3109/10253899709013744

[12] Luo E, Stephens SB, Chaing S, Munaganuru N, Kauffman AS, Breen KM. Corticosterone blocks ovarian cyclicity and the LH surge via decreased kisspeptin neuron activation in female mice. Endocrinology. 2016;157(3):1187‐1199. 26697722 10.1210/en.2015-1711 PMC4769373

[13] Dufourny L, Skinner DC. Type II glucocorticoid receptors in the ovine hypothalamus: distribution, influence of estrogen and absence of co-localization with GnRH. Brain Res. 2002;946(1):79‐86. 12133597 10.1016/s0006-8993(02)02829-9

[14] Ducret E, Anderson GM, Herbison AE. RFamide-related peptide-3, a mammalian gonadotropin-inhibitory hormone ortholog, regulates gonadotropin-releasing hormone neuron firing in the mouse. Endocrinology. 2009;150(6):2799‐2804. 19131572 10.1210/en.2008-1623

[15] Johnson MA, Tsutsui K, Fraley GS. Rat RFamide-related peptide-3 stimulates GH secretion, inhibits LH secretion, and has variable effects on sex behavior in the adult male rat. Horm Behav. 2007;51(1):171‐180. 17113584 10.1016/j.yhbeh.2006.09.009 PMC1831848

[16] Anderson GM, Relf HL, Rizwan MZ, Evans JJ. Central and peripheral effects of RFamide-related peptide-3 on luteinizing hormone and prolactin secretion in rats. Endocrinology. 2009;150(4):1834‐1840. 19022888 10.1210/en.2008-1359

[17] Kirby ED, Geraghty AC, Ubuka T, Bentley GE, Kaufer D. Stress increases putative gonadotropin inhibitory hormone and decreases luteinizing hormone in male rats. Proc Natl Acad Sci U S A. 2009;106(27):11324‐11329. 19541621 10.1073/pnas.0901176106 PMC2698887

[18] Geraghty AC, Muroy SE, Zhao S, Bentley GE, Kriegsfeld LJ, Kaufer D. Knockdown of hypothalamic RFRP3 prevents chronic stress-induced infertility and embryo resorption. Elife. 2015;4:e04316. 25581095 10.7554/eLife.04316 PMC4289855

[19] Yang JA, Song CI, Hughes JK, et al Acute psychosocial stress inhibits LH pulsatility and kiss1 neuronal activation in female mice. Endocrinology. 2017;158(11):3716‐3723. 28973125 10.1210/en.2017-00301 PMC5695836

[20] Yang JA, Hughes JK, Parra RA, Volk KM, Kauffman AS. Stress rapidly suppresses in vivo LH pulses and increases activation of RFRP-3 neurons in male mice. J Endocrinol. 2018;239(3):339‐350. 30382693 10.1530/JOE-18-0449 PMC6214202

[21] Leon S, Garcia-Galiano D, Ruiz-Pino F, et al Physiological roles of gonadotropin-inhibitory hormone signaling in the control of mammalian reproductive axis: studies in the NPFF1 receptor null mouse. Endocrinology. 2014;155(8):2953‐2965. 24823392 10.1210/en.2014-1030

[22] Mamgain A, Sawyer IL, Timajo DAM, et al RFamide-related peptide neurons modulate reproductive function and stress responses. J Neurosci. 2021;41(3):474‐488. 33219002 10.1523/JNEUROSCI.1062-20.2020 PMC7821868

[23] Sawyer IL, Evans MC, Mamgain A, Decourt C, Iremonger KJ, Anderson GM. Chemogenetic activation of RFRP neurons reduces LH pulse frequency in female but not male mice. J Endocr Soc. 2024;8(11):bvae159. 39381686 10.1210/jendso/bvae159 PMC11458915

[24] Poling MC, Luo EY, Kauffman AS. Sex differences in steroid receptor coexpression and circadian-timed activation of kisspeptin and RFRP-3 neurons may contribute to the sexually dimorphic basis of the LH surge. Endocrinology. 2017;158(10):3565‐3578. 28938464 10.1210/en.2017-00405 PMC5659694

[25] Bronson FH . The regulation of luteinizing hormone secretion by estrogen: relationships among negative feedback, surge potential, and male stimulation in juvenile, peripubertal, and adult female mice. Endocrinology. 1981;108(2):506‐516. 7449740 10.1210/endo-108-2-506

[26] Christian CA, Mobley JL, Moenter SM. Diurnal and estradiol-dependent changes in gonadotropin-releasing hormone neuron firing activity. Proc Natl Acad Sci U S A. 2005;102(43):15682‐15687. 16230634 10.1073/pnas.0504270102 PMC1257388

[27] Dror T, Franks J, Kauffman AS. Analysis of multiple positive feedback paradigms demonstrates a complete absence of LH surges and GnRH activation in mice lacking kisspeptin signaling. Biol Reprod. 2013;88(6):146. 23595904 10.1095/biolreprod.113.108555 PMC4070868

[28] Mohr MA, Esparza LA, Steffen P, Micevych PE, Kauffman AS. Progesterone receptors in AVPV kisspeptin neurons are sufficient for positive feedback induction of the LH surge. Endocrinology. 2021;162(11):bqab161. 34379733 10.1210/endocr/bqab161 PMC8423423

[29] Semaan SJ, Kauffman AS. Daily successive changes in reproductive gene expression and neuronal activation in the brains of pubertal female mice. Mol Cell Endocrinol. 2015;401:84‐97. 25498961 10.1016/j.mce.2014.11.025 PMC4312730

[30] Poling MC, Kauffman AS. Sexually dimorphic testosterone secretion in prenatal and neonatal mice is independent of kisspeptin-Kiss1r and GnRH signaling. Endocrinology. 2012;153(2):782‐793. 22202164 10.1210/en.2011-1838 PMC3275395

[31] Buch T, Heppner FL, Tertilt C, et al A Cre-inducible diphtheria toxin receptor mediates cell lineage ablation after toxin administration. Nat Methods. 2005;2(6):419‐426. 15908920 10.1038/nmeth762

[32] Ancel C, Inglis MA, Anderson GM. Central RFRP-3 stimulates LH secretion in male mice and has cycle stage-dependent inhibitory effects in females. Endocrinology. 2017;158(9):2873‐2883. 28475692 10.1210/en.2016-1902

[33] McEwen HJ, Inglis MA, Quennell JH, Grattan DR, Anderson GM. Deletion of suppressor of cytokine signaling 3 from forebrain neurons delays infertility and onset of hypothalamic leptin resistance in response to a high caloric diet. J Neurosci. 2016;36(27):7142‐7153. 27383590 10.1523/JNEUROSCI.2714-14.2016 PMC4938861

[34] Quennell JH, Howell CS, Roa J, Augustine RA, Grattan DR, Anderson GM. Leptin deficiency and diet-induced obesity reduce hypothalamic kisspeptin expression in mice. Endocrinology. 2011;152(4):1541‐1550. 21325051 10.1210/en.2010-1100 PMC3206710

[35] Steyn FJ, Wan Y, Clarkson J, Veldhuis JD, Herbison AE, Chen C. Development of a methodology for and assessment of pulsatile luteinizing hormone secretion in juvenile and adult male mice. Endocrinology. 2013;154(12):4939‐4945. 24092638 10.1210/en.2013-1502 PMC5398599

[36] Piet R . Circadian and kisspeptin regulation of the preovulatory surge. Peptides. 2023;163:170981. 36842628 10.1016/j.peptides.2023.170981

[37] Schenker JG, Meirow D, Schenker E. Stress and human reproduction. Eur J Obstet Gynecol Reprod Biol. 1992;45(1):1‐8. 1618356 10.1016/0028-2243(92)90186-3

[38] Rizwan MZ, Poling MC, Corr M, et al RFamide-related peptide-3 receptor gene expression in GnRH and kisspeptin neurons and GnRH-dependent mechanism of action. Endocrinology. 2012;153(8):3770‐3779. 22691552 10.1210/en.2012-1133

[39] Breen KM, Billings HJ, Wagenmaker ER, Wessinger EW, Karsch FJ. Endocrine basis for disruptive effects of cortisol on preovulatory events. Endocrinology. 2005;146(4):2107‐2115. 15625239 10.1210/en.2004-1457

[40] Wang L, Vanacker C, Burger LL, et al Genetic dissection of the different roles of hypothalamic kisspeptin neurons in regulating female reproduction. Elife. 2019;8:e43999. 30946012 10.7554/eLife.43999 PMC6491090

[41] Mitchell JC, Li XF, Breen L, Thalabard JC, O'Byrne KT. The role of the locus coeruleus in corticotropin-releasing hormone and stress-induced suppression of pulsatile luteinizing hormone secretion in the female rat. Endocrinology. 2005;146(1):323‐331. 15486230 10.1210/en.2004-1053

[42] Evans MC, Anderson GM. The role of RFRP neurons in the allostatic control of reproductive function. Int J Mol Sci. 2023;24(21):15851. 37958834 10.3390/ijms242115851 PMC10648169

